# Bioreactors for Decreasing the Growth of Brain Tumors

**DOI:** 10.1100/tsw.2001.25

**Published:** 2001-05-01

**Authors:** Rona S. Carroll, Marcelle Machluf

**Affiliations:** Brigham and Women's Hospital, Boston, MA 02115, USA

**Keywords:** glioma, endostatin, angiogenesis

## Abstract

Malignant gliomas are the most common primary brain tumors. They are highly aggressive tumors characterized by a recurrence rate of virtually 100%. Despite significant advances in neuroimaging and neurosurgical techniques, the median survival time of patients with glioblastoma multiforme remains 12 to 18 months. Malignant gliomas are characterized by rapidly dividing cells, which invade into the normal brain, and a high degree of vascularity. Recent experimental evidence indicates that tumor-related angiogenesis contributes significantly to the malignant phenotype.

To date, several inhibitors that prevent blood vessel formation (angiogenesis) have been isolated and tested for their ability to inhibit brain tumor growth including platelet factor 4, TNP-40, heparin, penicillamine, hydrocortisone, and angiostatin [1-7]. Recently, O'Reilly et al. [8] identified a novel endogenous specific inhibitor of tumor-related endothelial cell proliferation, endostatin. Endostatin is a 20 kDa cleavage product of the C-terminal domain of collagen XVIII and was purified from murine hemangioendothelioma cells.

In the January *Nature Biotechnology* [9], our laboratory published a manuscript that describes the successful microencapsulation of producer cells genetically engineered to secrete biologically active endostatin. These capsules were implanted around a human-derived glioma tumor grown in nude mice. For 21 days, these tiny "bioreactors" lived inside the mouse, continuously secreting endostatin while inhibiting the tumor's growth without provoking an immune response.

The capsules are made from alginate and are further coated with another polymer poly L-(lysine). The combination of these two polymers creates a membrane that shields the endostatin-secreting cells from the immune system while allowing nutrients in and endostatin out. The preparation procedure takes place in aqueous solutions that use mild salts, avoiding possible damage to the cells and the endostatin they secrete.

The release of biologically active endostatin was confirmed using a bovine capillary endothelial (BCE) proliferation assay and tube formation assay. In the *in vivo* model a single local injection of encapsulated endostatin-secreting cells resulted in a reduction of subcutaneous U87 xenografts weight by 72.3% 21 days post-treatment in a mice model.

Endostatin is a specific inhibitor of endothelial cell proliferation and is a potent angiogenic inhibitor. Achieving concentrations of endostatin sufficient to combat tumor growth is complicated by the exceedingly short half-life of the protein (30 min; personal communication). Reaching and maintaining therapeutically efficacious levels of endostatin may be achieved either by continuous or repeated injections, or by a mechanism whereby the protein is continuously secreted by an implanted device. In work done by O'Reilly et al. [8], daily systemic administration of 2.5 mg/kg endostatin for more than 16 days resulted in a 53% inhibition in tumor growth in a Lewis lung carcinoma model. In a renal cell carcinoma model, recombinant human endostatin injected daily subcutaneously (10 μg/kg/day) around the tumor for 4 days led to a 61% inhibition of tumor growth after 20 days. Intramuscular injection of naked mouse endostatin expression plasmid combined with polyvinylpyrrolidone (Pladsone®) once a week for a period of two weeks led to a 20% inhibition of tumor growth 18 days post-treatment [10]. The present study demonstrates that a single administration of microencapsulated-engineered BHK cells that continuously secrete an average endostatin concentration of 150.8 ng/ml/week inhibits the glioma tumors in mice. With use of this system, lower doses of the effective antiangiogenic protein may achieve significant tumor suppression.

The lab's next experimental step is trying to implant endostatin bioreactors at the site of an intracranial tumor. This work, with microencapsulated endostatin inside mice, is a long way from Folkman's clinical trials using exogenous endostatin in human cancer patients. Those trials are proceeding in Boston, Houston, and Madison, Wisconsin. Patients with malignant glioma of the brain were specifically excluded from the first human trials so endostatin's effect on gliomas is still to be explored.

This type of therapy may be one of the future biological therapies for these tumors. Chemotherapy and surgery has not proved to be the ultimate solution for this type of tumor. The encapsulation cell therapy is very much a work in progress, but definitely this is a concept that will be pursued further in the future.

| | | |
|---|---|---|
| Received: | February | 8, 2001 |
| Accepted: | March | 26, 2001 |
| Published: | May | 1, 2001 |
